# Supplementary material for: Molecular Determinants of Fibrillation in a Viral Amyloidogenic Domain from Combined Biochemical and Biophysical Studies
Source: Int J Mol Sci. 2022 Dec 26;24(1):399. doi: 10.3390/ijms24010399 (PMC9820236; doi:10.3390/ijms24010399)
Supplement: Supplementary file 1 [file ijms-24-00399-s001.zip › ijms-2072609-supplementary.pdf]

Table S1. Analysis of physicochemical properties of full-length PNT3 *wt*, PNT3 truncated and C-terminal region as provided by CIDER (http://pappulab.wustl.edu/CIDER/, accessed on 24 October 2022) [1].

	**PNT3 *wt***	**PNT3_trunc**	**C-terminal**
**N**	133	77	56
**f-**	0.22556	0.19481	0.26786
**f+**	0.09774	0.09091	0.10714
**FCR**	0.32331	0.28571	0.37500
**NCPR**	-0.12782	-0.10390	-0.16071
**Kappa**	0.25641	0.36530	0.17898
**Hydropathy**	3.27594	3.25584	3.30357
**Phase Plot Region**	2	2	3
**Phase Plot Annotation**	Boundary Region	Boundary region	Coils,Hairpins and Chimeras
**Net charge at pH 7.0**	-16.34	-7.44	

N: Number of residues; **f-:** Fraction of negatively charged residues; **f+:** Fraction of positively charged residues**;** **FCR:** Fraction of charged residues; **NCPR:** Net charge per residue; **Kappa:** κ (charge patterning parameter); **Hydropathy:** The 0-9 scaled Kyte-Doolittle hydropathy score for the sequence (9 most hydrophobic, 0 least hydrophobic); **Phase Plot Region:** Location on the Das-Pappu phase plot this sequence falls; **Phase Plot Annotation:** Annotation associated with a specific region of the Das-Pappu phase plot.

Table S2. Nucleotide sequence of the primers used to generate the various constructs.

Primer Name	Sequence (5'-3')	Name of the consturct	Reference
HeV PNT3-AttB1	GGGGACAAGTTTGTACAAAAAAGCAGGCTTCACCCCGACCGAAGAACCGCCG	PNT3-pDEST17	Salladini, et al., 2021
HeV PNT3-AttB2	GGGGACCACTTTGTACAAGAAAGCTGGGTCTTATTAGCTATCTTTACGACGCACATC		
attB1	ACAAGTTTGTACAAAAAAGCAGGCT		
attB2	ACCACTTTGTACAAGAAAGCTGGGT		
R_3ala-PNT3	TCGCCACGACGGCCGCTACCGGCTGCCGCTTCCGGAATAACCGGCGGTTCTTC	PNT3^{3A}-pDEST17	Salladini, et al., 2022
F_3ala-PNT3	AACCGCCGGTTATTCCGGAAGCGGCAGCCGGTAGCGGCCGTCGTGGCGATCTG		
F_ala1_PNT3	CGGAAGCGTATTATGGTAGCGGCCGTCGTGGCGA	PNT3^{A1}-pDEST17	This work
R_ala1_PNT3	GCTACCATAATACGCTTCCGGAATAACCGGCGGTT		
F_ala2_PNT3	CGGAATATGCGTATGGTAGCGGCCGTCGTGGCGA	PNT3^{A2}-pDEST17	This work
R_ala2_PNT3	GCTACCATACGCATATTCCGGAATAACCGGCGGTT		
F_ala3_PNT3	CGGAATATTATGCGGGTAGCGGCCGTCGTGGCGA	PNT3^{A3}-pDEST17	This work
R_ala3_PNT3	GCTACCCGCATAATATTCCGGAATAACCGGCGGTT		
NiV PNT3-AttB1	ACAAGTTTGTACAAAAAAGCAGGCTCCGATCCTGCAAAAGACTCTCC	NiV_PNT3-pDEST17	This work
NiV PNT3-AttB2	ACCACTTTGTACAAGAAAGCTGGGTTTATTATGAGTCCTTTGGACCGGCAC		
Trunc_PNT3_B2	ACCACTTTGTACAAGAAAGCTGGGTCTTATTAAAATTCATCTTCATATTCCAG	PNT3_C-term_truncated - pDEST17	This work
PNT3_C255_F	CTGGAATATGAAGATGAATTTTGCAAAAGCAGCAGCGAAGTGGTG	PNT3_Cys-pDEST17	This work
PNT3_C255_R	AAATTCATCTTCATATTCCAG		

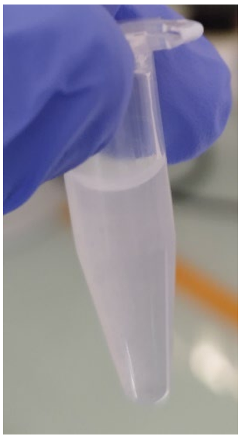

Figure S1. Cloudy solution of the HeV PNT3 protein obtained after exchanging the buffer with a Sephadex G-25 medium column previously equilibrated with sodium phosphate 50 mM buffer at pH 4.0.

```
           1       10        20        30        40        50        60        70        80        90        100       110       120       130
           |--------+---------+---------+---------+---------+---------+---------+---------+---------+---------+---------+---------+---------|
      NiV  MDKLELVNDGLNIIDFIQKNQKEIQKTYGRSSIQQPSIKDQTKAWEDFLQCTSGESEQVEGGMSKDDGDVERRNLEDLSSTSPTDGTIGKRVSNTRDWAEGSDDIQLDPVVTDVVYHDHGGECTGYGFTS
 HeV-PNT3
Consensus  ..................................................................................................................................

           131     140       150       160       170       180       190       200       210       220       230       240       250       260
           |--------+---------+---------+---------+---------+---------+---------+---------+---------+---------+---------+---------+---------|
      NiV  SPERGWSDYTSGANNGNVCLVSDAKMLSYAPEIAVSKEDRETDLVHLENKLSTTGLNPTAVPFTLRNLSDPAKDSPVIAEHYYGLGVKEQNVGPQTSRNVNLDSIKLYTSDDEEADQLEFEDEFAGSSSE
 HeV-PNT3                                                                       TPTEEPPVIPEYYYGSGRRGDLSKSPPRGNVNLDSIKIYTSDDEDENQLEYEDEFAKSSSE
Consensus  .....................................................................dPae#pPVIaEhYYGlGrre#nsgpqprrNVNLDSIKiYTSDDE#a#QLE%EDEFAgSSSE

           261     270       280       290       300       310       320       330       340       350       360       370       380       390
           |--------+---------+---------+---------+---------+---------+---------+---------+---------+---------+---------+---------+---------|
      NiV  VIVGISPEDEEPSSVGGKPNESIGRTIEGQSIRDNLQAKDNKSTDVPGAGPKDSAVKEEPPQKRLPMLAEEFECSGSEDPIIRELLKENSLINCQQGKDAQPPYHWSIERSISPDKTEIVNGAVQTADRQ
 HeV-PNT3  VVIDTTPEDNDSIN----QEEVVGDPSDQGLEHPFPLGKFPEKEETPDVRRKDS
Consensus  V!!disPED##pin....q#Es!Grpi#qqlerdnlqaKdneke#tPdarrKDS............................................................................

           391     400       41012
           |--------+---------+-|
      NiV  RPGTPMPKSRGIPIKKHHHHHH
 HeV-PNT3
Consensus  ......................
```

Figure S2. Alignment of NiV PNT3 and HeV PNT3 regions.

NiV PNT3

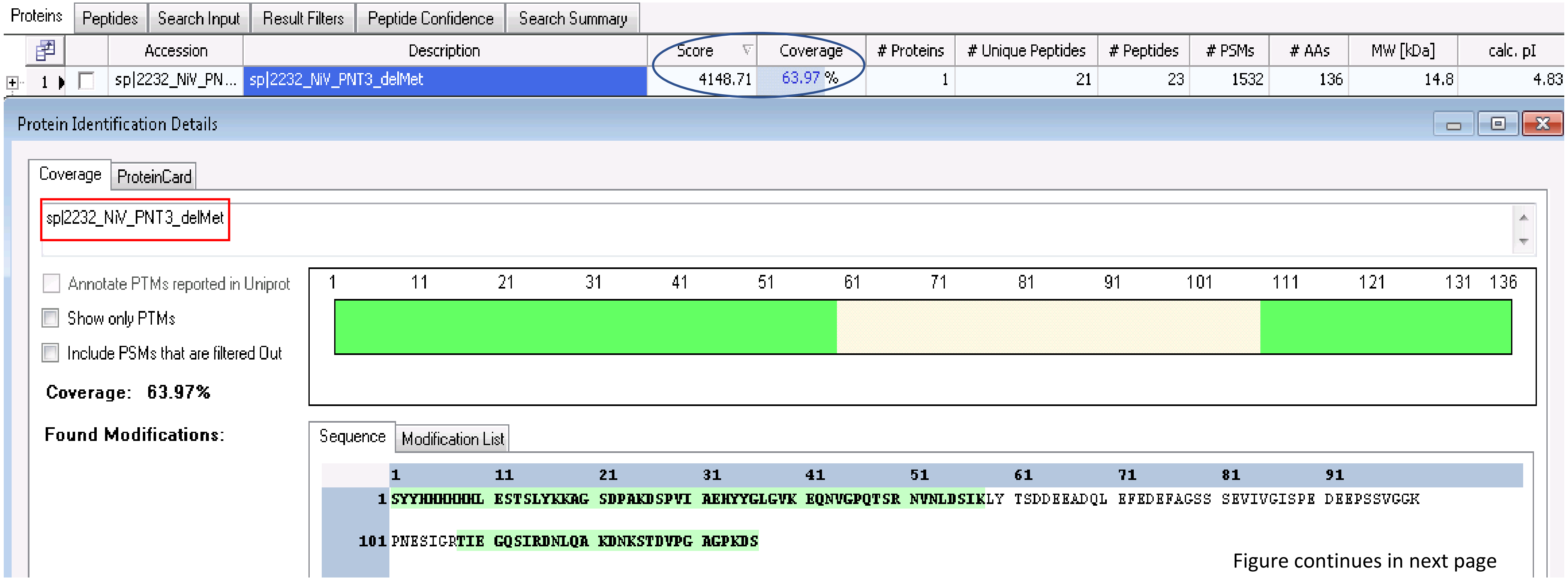

Figure continues in next page

HeV PNT3_Cys

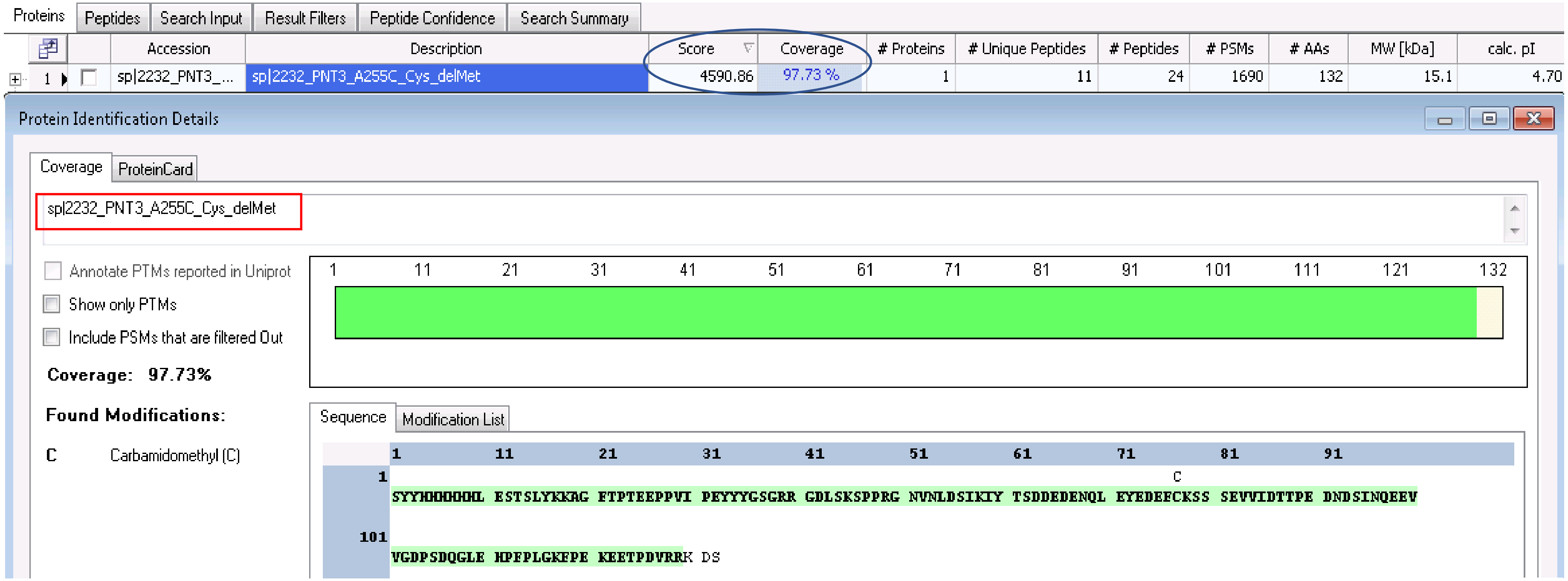

Figure continues in next page

HeV PNT3 C-terminal_truncated

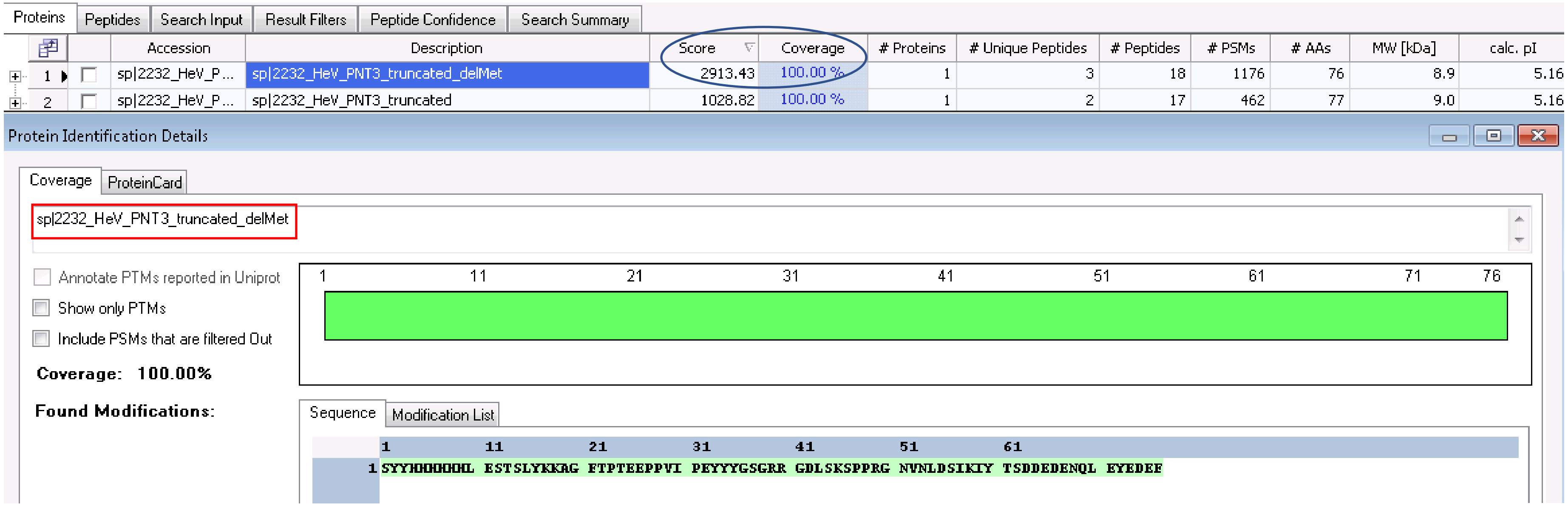

Figure continues in next page

HeV PNT3^{3A} C-terminal_truncated

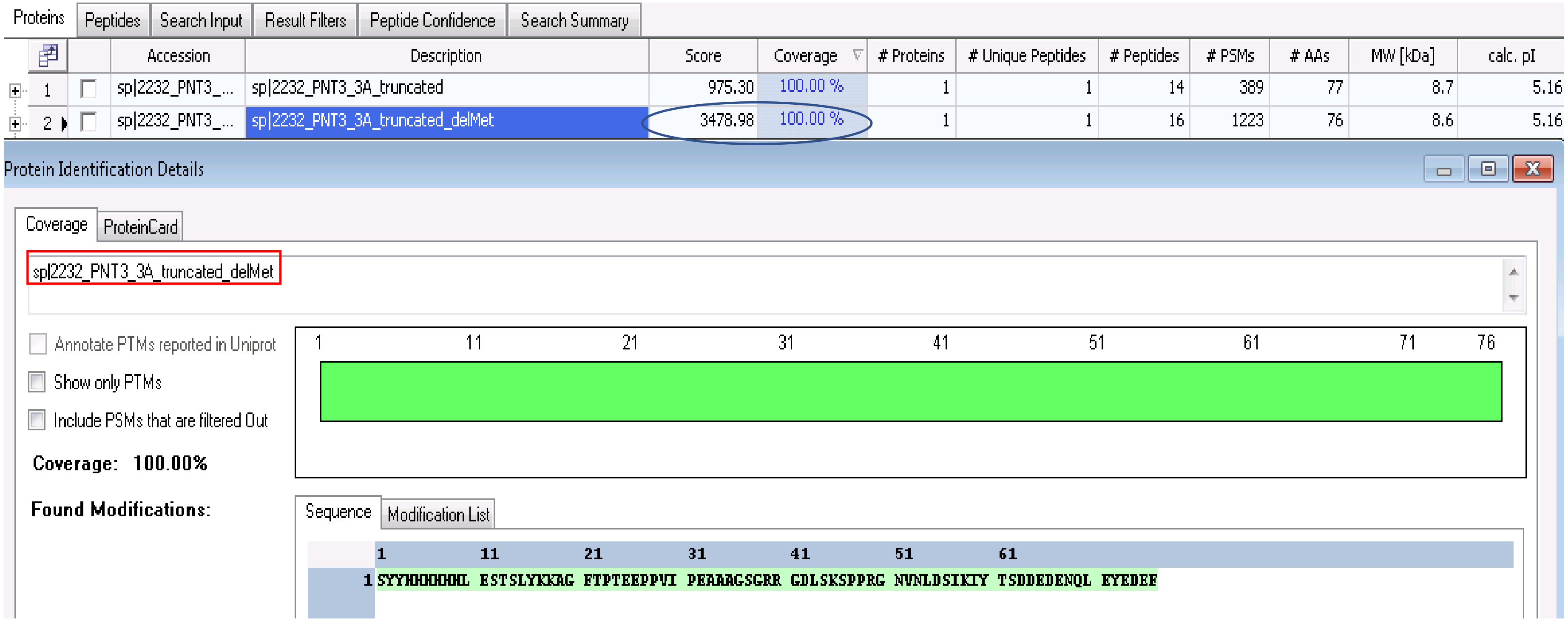

Figure continues in next page

HeV PNT3[A1]

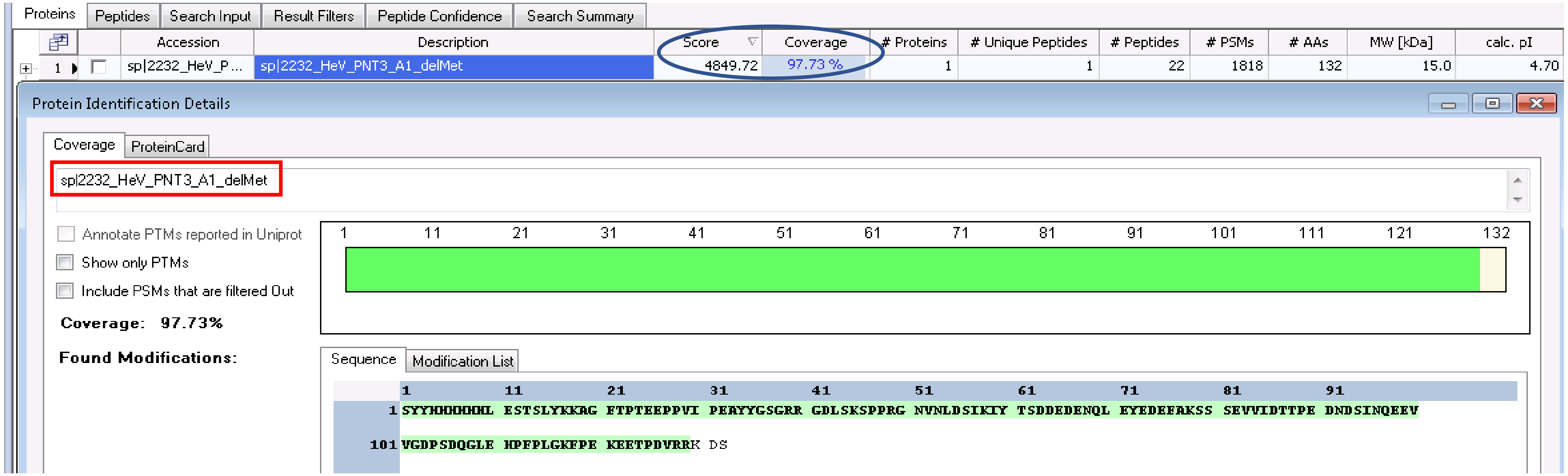

MSMS of the (19-39) specific peptide from the identification of the HEV_PNT3_A1 band

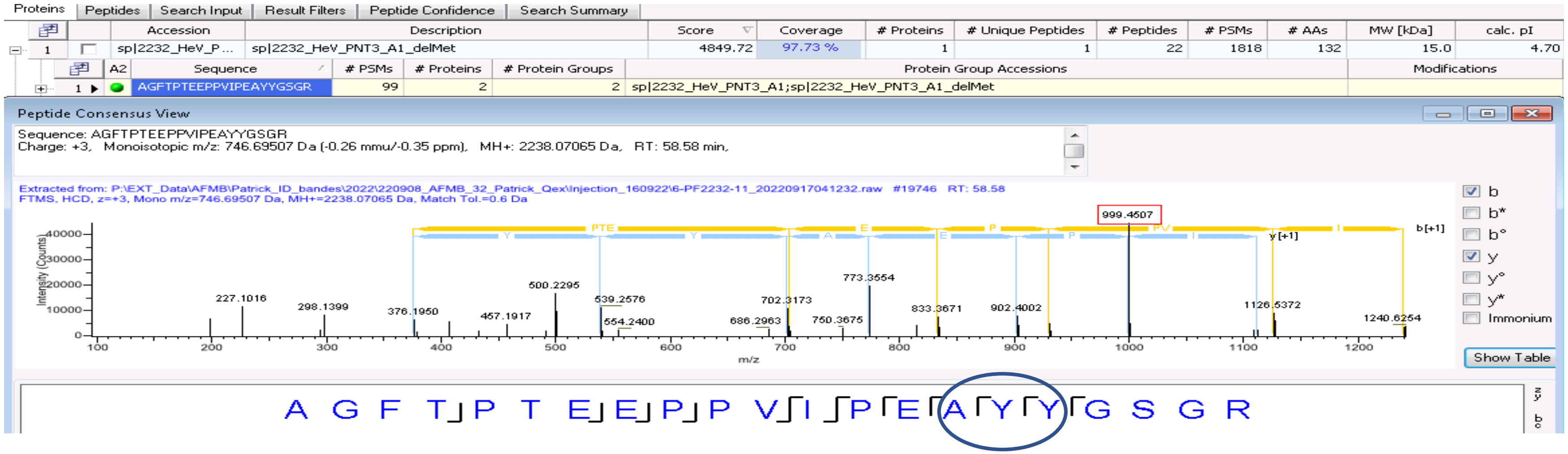

Figure continues in next page

HeV PNT3^{A2}

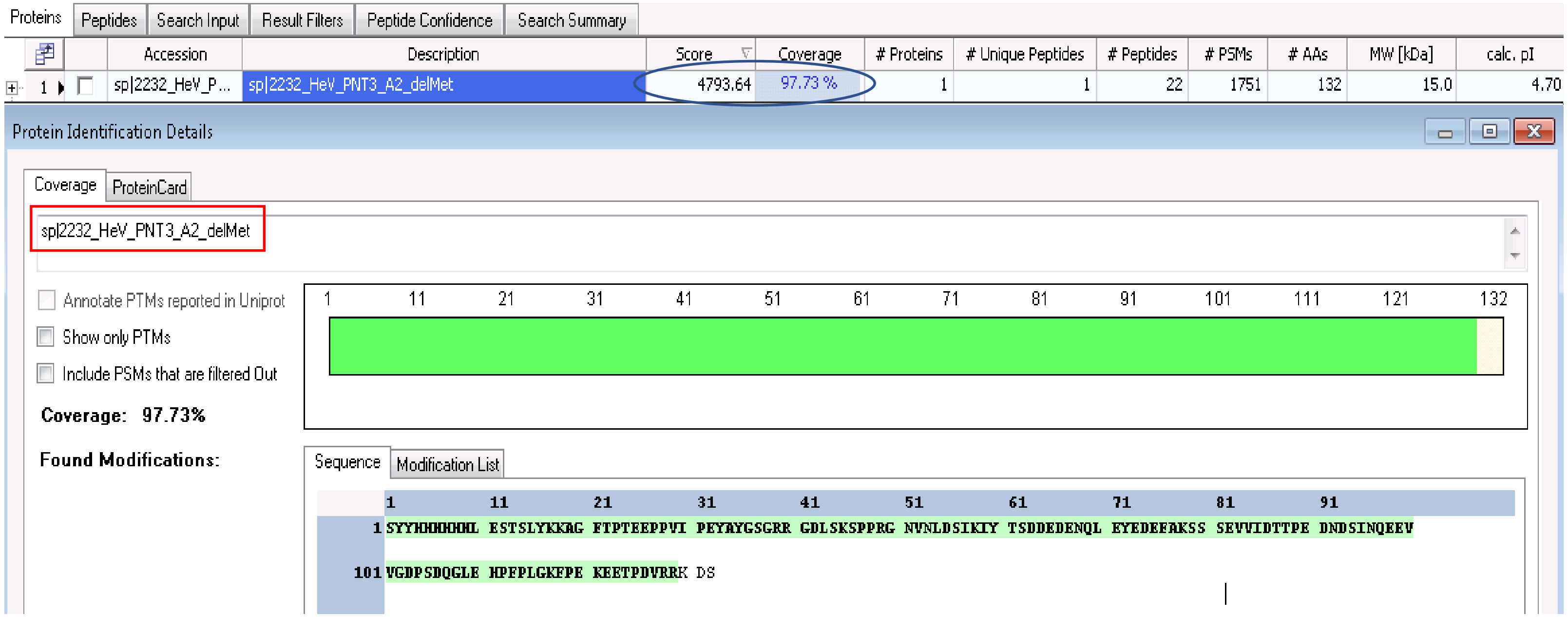

MSMS of the (19-39) specific peptide from the identification of the HEV_PNT3_A2 band

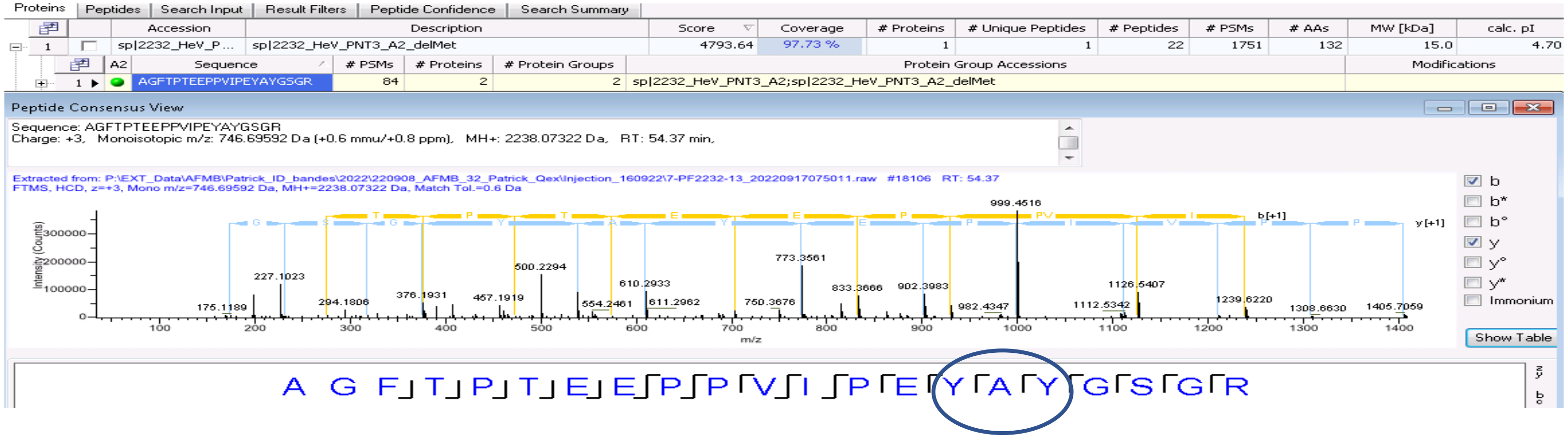

Figure continues in next page

HeV PNT3[A3]

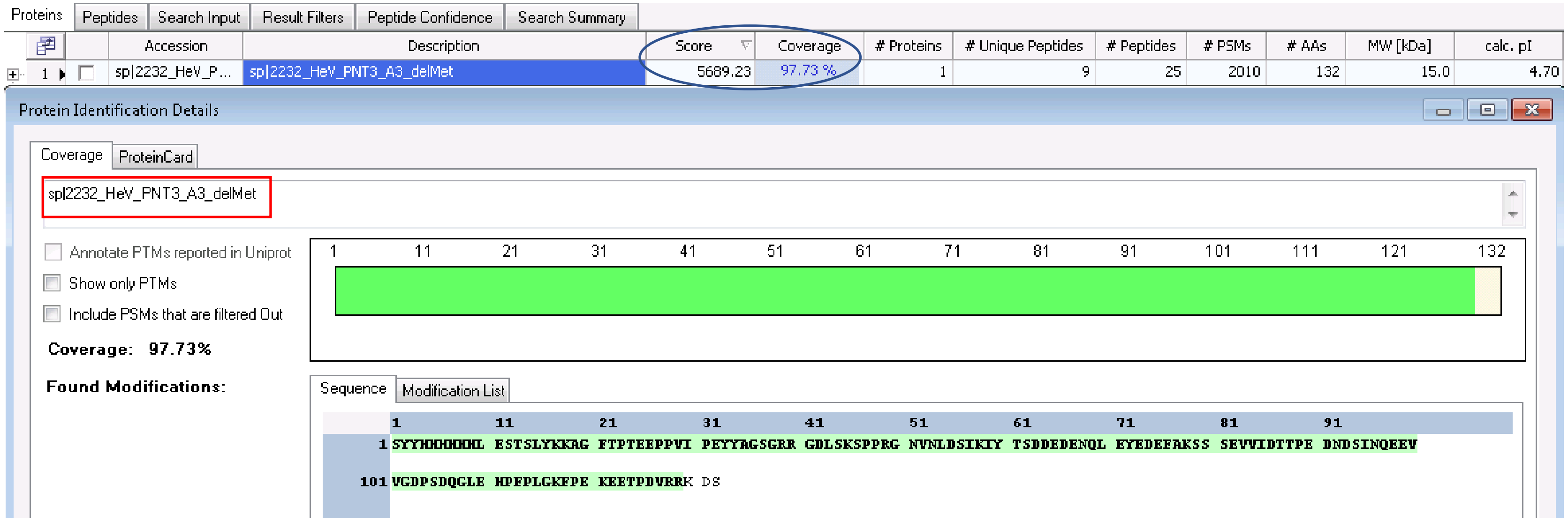

MSMS of the (19-39) specific peptide from the identification of the HEV_PNT3_A3 band

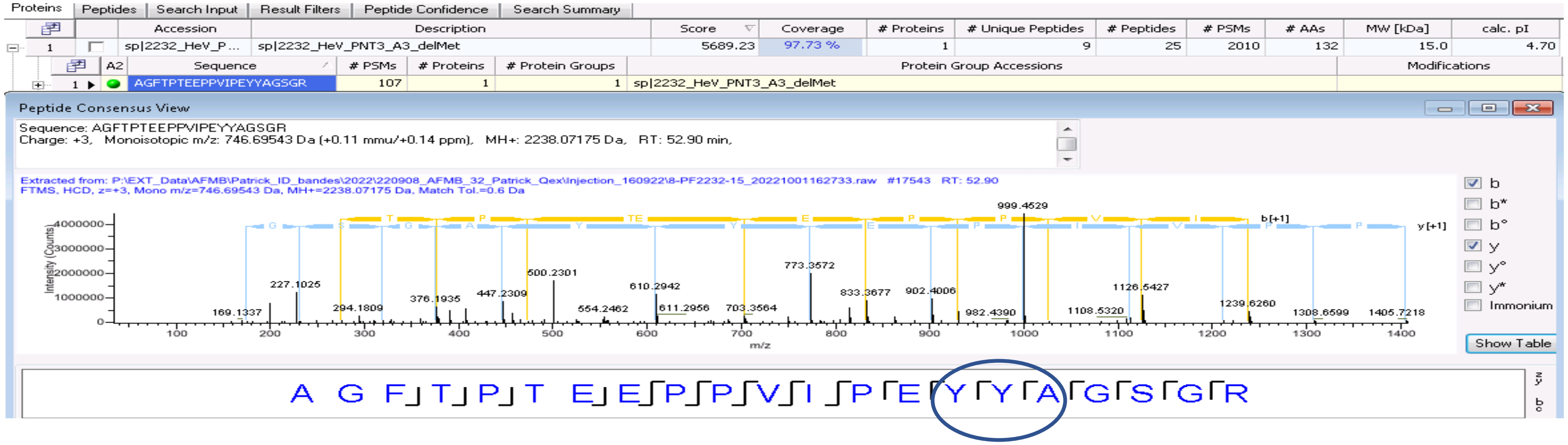

Figure S3. Mass Spectrometry (MS) results of the PNT3 variants after digestion of the purified protein bands excised from SDS-polyacrylamide gels. Mass spectrometry analyses were carried out by LC-MSMS using an Orbitrap Qexactive spectrometer online with a nanoLC Ultimate 3000 chromatography system. The peaks obtained have been compared to the theoretical digestion peak list of the expected protein. Expected peptides found in the spectrum are represented in green. The ID score and coverage are circled. In the case of variants PNT3^{A1}, PNT3^{A2} and PNT3^{A3}, the interpretation of the results was not straightforward as the sequences are very similar and a very thorough analysis of the MSMS spectra was required. For each band, a representative MSMS spectrum of the specific (18-39) peptide is presented showing with no ambiguity the exact sequence of the protein. All the "y" fragments (in blue) containing the right amino acids in the right sequence are seen and shown squared in blue.

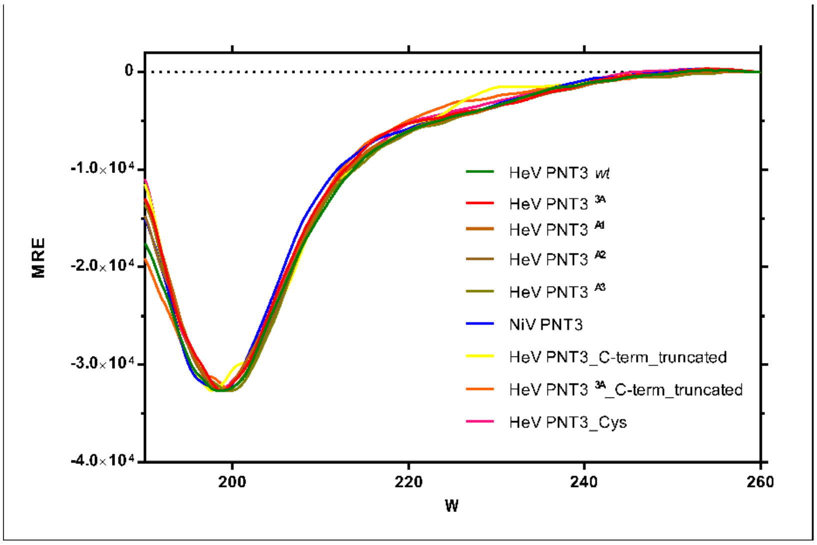

Figure S4. Far-UV circular dichroism (CD) studies of PNT3 variants. **A.** Spectra of PNT3 variants recorded in 10 mM sodium phosphate pH 7.2 at 37 °C. Protein concentration was 0.06 mg mL^{-1} (4 µM). Spectra were recorded from a freshly purified PNT3 variant sample recorded immediately after elution from the SEC column. MRE (Θ) is expressed in deg cm^2 $dmol^{-1}$.

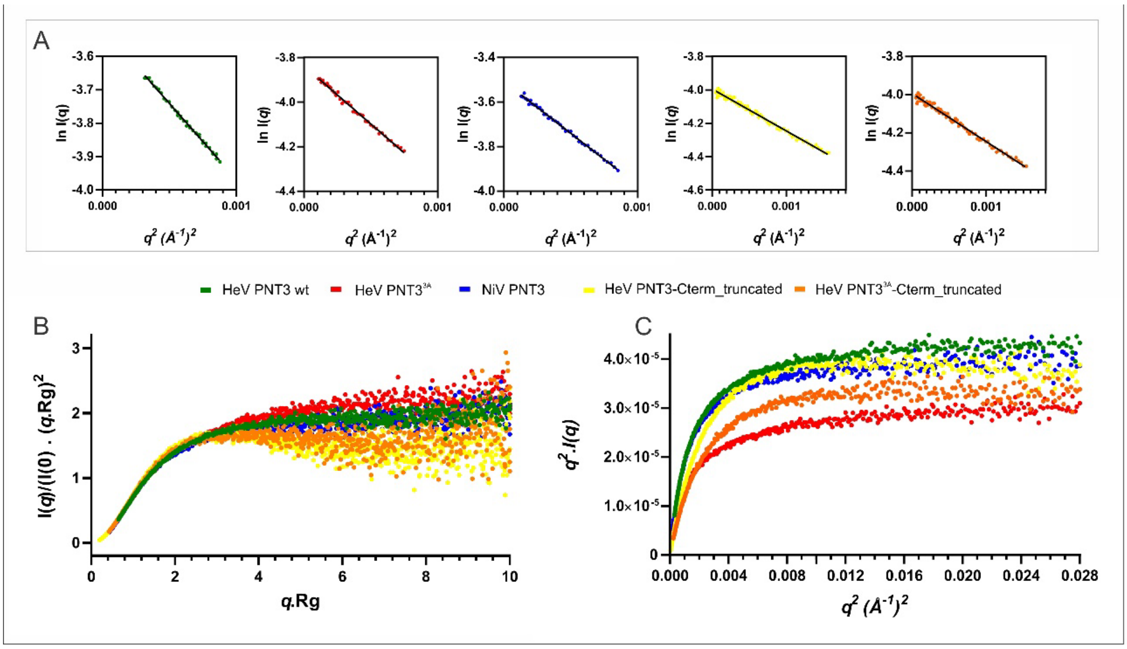

Figure S5. SEC-SAXS analysis of the monomeric form of PNT3 variants. **A.** Guinier plots of the experimental scattering curves used to determinate the radius of gyration (Guinier approximation at low angles (q_{max}.Rg < 1.1)). **B**. Normalized Kratky plot. **C.** Kratky-Debye plot.

*MSYYHHHHHH LESTSLYKKA GF*TPTEEPPV IPEYYYGSGR RGDLSKSPPRGNVNLDSIKI YTSDDEDENQ
LEYEDEF<u>AKS SSEVVIDTTP EDNDSINQEEVVGDPSDQGL EHPFPLGKFP EKEETPDVRR KDS</u>

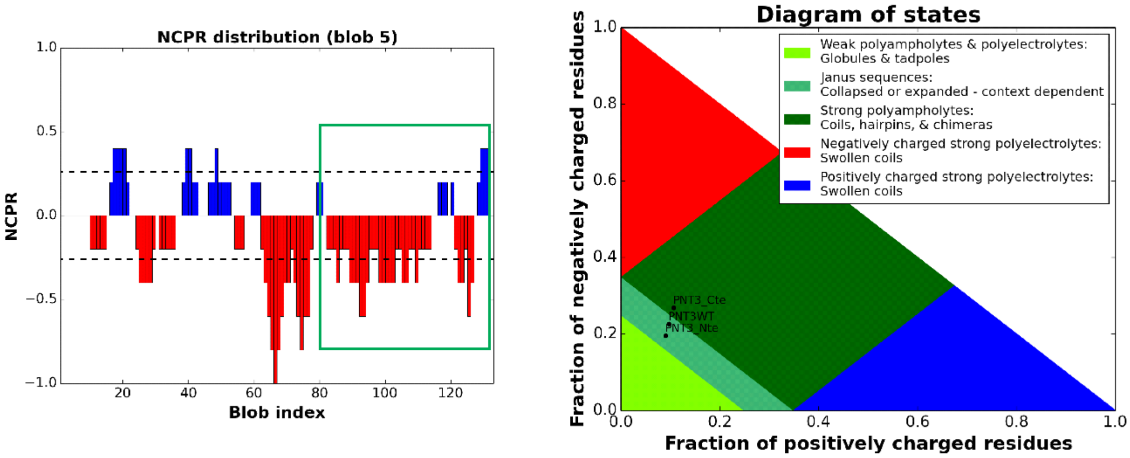

Figure S6. Analysis of charge distribution within the PNT3 sequence. The amino acid sequence of the recombinant protein is shown on the top, with vector-encoded residues shown in italic on a grey background, and residues removed in the PNT3 C-terminally truncated variant underlined. Left panel: net charge per residues (NCPR) as a function of residue number, with the C-terminal region framed. Right panel: phase diagram plot of full-length PNT3 *wt*, and of its N-terminal (Nte, aa 200-254) and C-terminal (Cte, aa 255-310) regions right panel) as provided by CIDER (http://pappulab.wustl.edu/CIDER/, accessed on 24 October 2022) [1].

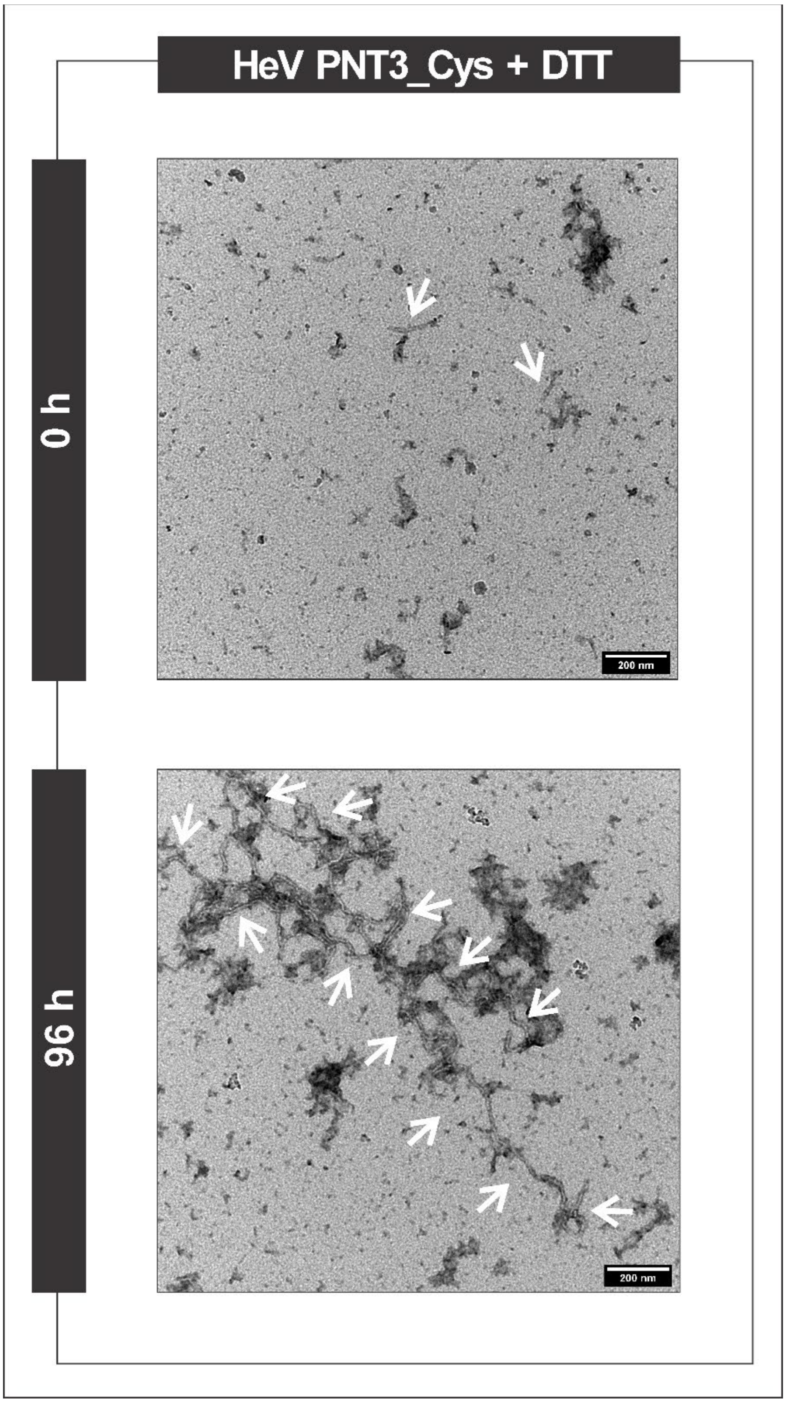

Figure S7. Ns-TEM analysis of HeV PNT3_Cys (200 μM) in the presence of DTT (10 mM) at time zero and after 96 h of incubation at 37 °C. Note that in all cases, samples were diluted to 40 μM prior deposition on the grid. The white arrows indicate fibrils.

References

1. Holehouse, A.S.; Das, R.K.; Ahad, J.N.; Richardson, M.O.; Pappu, R.V. CIDER: Resources to Analyze Sequence-Ensemble Relationships of Intrinsically Disordered Proteins. *Biophysical journal* 2017, *112*, 16-21, doi:10.1016/j.bpj.2016.11.3200.
